# The influence of corneal astigmatism on retinal nerve fiber layer thickness and optic nerve head parameter measurements by spectral-domain optical coherence tomography

**DOI:** 10.1186/1746-1596-7-55

**Published:** 2012-05-23

**Authors:** Lin Liu, Jun Zou, Hui Huang, Jian-guo Yang, Shao-rong Chen

**Affiliations:** 1 Department of Ophthalmology, Shanghai Jiaotong University Affiliated Sixth People’s Hospital, Shanghai, 200233, China

**Keywords:** High myopia, Corneal ASTIGMATISM, Optical coherence tomography

## Abstract

**Background:**

To evaluate the influence of corneal astigmatism (CA) on retinal nerve fiber layer (RNFL) thickness and optic nerve head(ONH) parameters measured with spectral-domain optical coherence tomography (OCT) in high myopes patients before refractive surgery.

**Methods:**

Seventy eyes of 35 consecutive refractive surgery candidates were included in this study. The mean age of the subjects was 26.42 ± 6.95 years, the average CA was −1.17 diopters (D; SD 0.64; range −0.2 to-3.3D), All subjects in this study were WTR CA. 34 eyes were in the normal CA group with a mean CA was −0.67 ± 0.28D, 36 eyes were in the high CA group with an average CA of −1.65 ± 0.49D. All subjects underwent ophthalmic examination and imaging with the Cirrus HD OCT.

**Results:**

No significant difference was noted in the average cup-to-disk ratio, vertical cup-to-disk ratio and cup volume (all *P* values > 0.05). Compared with the normal CA group, the high CA group had a larger disc area and rim area, thinner RNFL thickness in the temporal quadrant, and the superotemporal and inferotemporal peaks were farther to the temporal horizon (All *P* values < 0.05). There were no significant differences between the two groups in global average RNFL thickness, as well as superior, nasal and inferior quadrant RNFL thickness (all *P* values > 0.05).

**Conclusions:**

The degree of with-the-rule CA should be considered when interpreting ONH parameters and peripapillary RNFL thickness measured by the Cirrus HD OCT.

**Virtual slides:**

The virtual slide(s) for this article can be found here: http://www.diagnosticpathology.diagnomx.eu/vs/1148475676881895

## Introduction

Astigmatism is a worldwide common ocular disorder. Total astigmatism is mainly driven by corneal astigmatism(CA), which occurs due to an irregular shape of the cornea. In eyes with astigmatism, retinal images can be distorted. Langenbucher et al. [1] reported that the retinal image was distorted to an ellipse, and the image size could vary according to the axis of astigmatism assessed with computer-based methodology in astigmatic eyes.

Optical coherence tomography (OCT) can provide imaging of ocular structures by a noninvasive method, It is widely used in clinical and scientific ophthalmology to obtain high-resolution cross-sections of the retina images. The thickness of the retinal nerve fiber layer (RNFL) and optic nerve head (ONH) parameters can be measured by OCT. Evaluation of these parameters is essential, since the thickness of the RNFL may be effected in various diseases. For example, the RNFL becomes thinner in glaucoma and optic atrophy, whereas it is thicker in papilledema.

Many studies have reported the effect of refractive error changes induced by refractive surgery or contact lenses on RNFL thickness measured by OCT [2-4], while little is known about the effect of cylindrical refractive error (astigmatism) on RNFL and ONH parameters measured by OCT. The purpose of this study was to evaluate the influence of corneal astigmatism on the peripapillary RNFL thickness and ONH parameters obtained by Cirrus HD spectral-domain OCT (Cirrus HD OCT; Carl Zeiss Meditec, Dublin, CA, USA) in Chinese subjects with high myopia.

## Material and methods

### Subjects

70 eyes of 35 consecutive refractive surgery candidates with spherical equivalent ≥ −6 diopters (D) were recruited for the study. Ethical approval for the study was obtained from the local medical ethics committee. All subjects were volunteers and informed consents were obtained.

**Figure 1:**
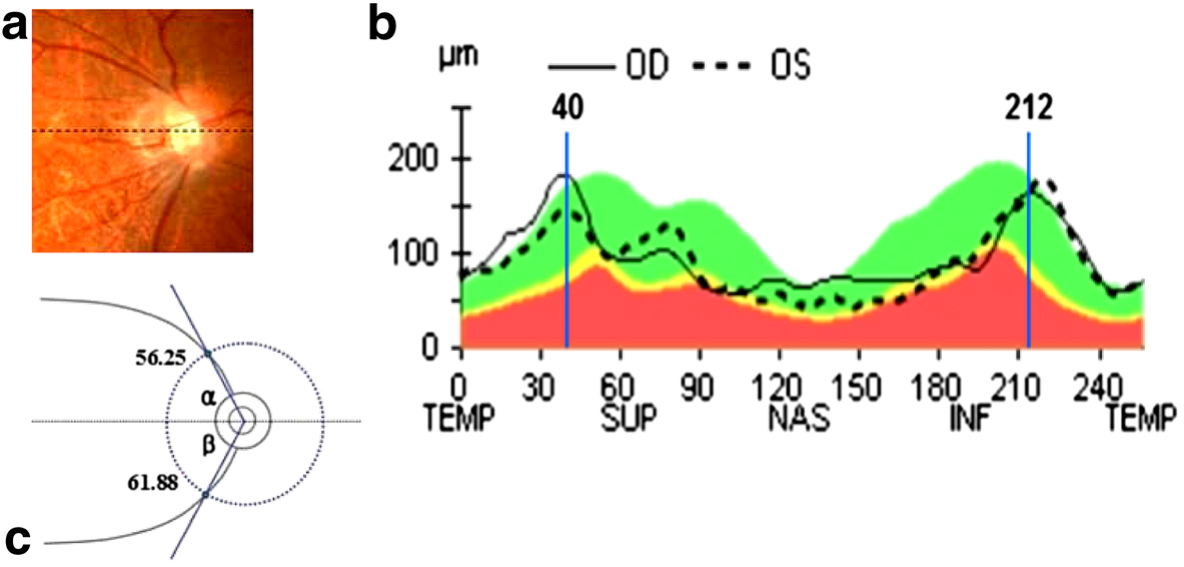
**An example of a measurement of the retinal nerve fiber layer characteristics in an right eye with a-6.625D of spherical equivalent, a -1.6 of CA and a 26.42mm of axial length: (a) Fundus photograph of the optic disc.** Dotted line represents imaginary horizontal meridian; **(b)** the peak locations at the superior and inferior area were 40 and 212, respectively; **(c)** the peak locations were translated to units of degrees by multiplying 360/256. Angles between the horizontal meridian and the superotemporal / inferotemporal peak locations were defined as the α (superotemporal) and β (inferotemporal) angles, so RNFL peak locations of this eye were α = 40 × 360/256 = 56.25(degree), β = 360-212 × 360/256 = 61.88(degree).

Each subject underwent a full ophthalmic examination, which included measures of visual acuity, refraction, intraocular pressure (IOP) by a noncontact tonometer. Axial length measurements were obtained in each eye with the IOL Master (Carl Zeiss Meditec, Inc, Dublin, CA), CA measurements were obtained by a Topolyzer (Allegretto Wave Topolyzer, Germany), optic nerve head evaluation was performed with a 90-D lens, and peripapillary RNFL thickness and ONH parameters were measured with the Cirrus HD OCT (Cirrus HD OCT; Carl Zeiss Meditec, Dublin, CA). The peak locations of the superotemporal and inferotemporal areas were evaluated by the RNFL TSNIT curve of the Cirrus HD OCT. The peak locations, which were measured by the RNFL TSNIT curve, were translated to units of degrees by multiplying 360/256. For example, the superior peak location of 40 in the TSNIT curve was translated to 56.25 degrees (40 × 360/256 degrees). This means that the thickest superior RNFL was located at the point 56.25 degrees away from the temporal horizontal meridian. We defined α angle as the angle between the horizontal meridian and superotemporal peak location by clockwise rotation, and the angle between the horizontal meridian and inferotemporal peak locations by counterclockwise rotation were defined as β angle (Figure 1).

The individuals were included if they had the following: best corrected visual acuity of 20/20 or better, an intraocular pressure (IOP) lower than 21 mmHg in either eye, CA as a with-the-rule (WTR) astigmatism, a healthy ONH without glaucomatous damage (i.e., no disc haemorrhage, notching or thinning of the neural rim).

Those with a history of severe ocular trauma, intraocular or refractive surgery or any ocular or neurological disease that could have affected the ONH or RNFL were excluded from the study. Subjects with evidence of macular disease or peripapillary atrophy extending more than 1.73 mm from the center of the optic disc or with glaucoma or an IOP higher than 21 mmHg in either eye were also excluded. In addition, participants with a history of systemic diseases including hypertension and diabetes were excluded.

We assigned astigmatic types as defined in Katz and Kruger [5]: with-the-rule (WTR) astigmatism was assigned if the plus cylinder axis was within 30° of 90°, against-the-rule (ATR) astigmatism was assigned if the plus cylinder axis was within 30° of 180°, and the others were assigned as oblique. Astigmatism was defined as equal to 1.0 D, as in multiple previous studies [6,7].

### Astigmatism

CA was measured with Topolyzer (Allegretto Wave Topolyzer, Germany) and the total astigmatism was measured by a refractor keratometer (Topcon KR-8800 Auto Refractor).

### OCT imaging

After pupillary dilation to a minimum diameter of 5 mm, the eyes of the subjects that satisfied the study criteria were scanned using the Cirrus HD-OCT system with software version 5.0. All the scans had signal strength of at least 6 and all measurements were taken by a single, well-trained examiner. The superior clock hour was 12 o’clock and the others were assigned accordingly in a clockwise manner in the right eye and counterclockwise in the left eye.

### Statistical analysis

Statistical analyses were performed with commercially available software (SPSS ver. 17.0; SPSS Inc, Chicago, IL). The total average and mean clock hour RNFL measurements were compared between the two groups with an independent *t*-test. Correlations between RNFL parameters and astigmatism were examined by linear regression analysis and expressed as the Pearson coefficient of correlation (*r*). A p value <0.05 was considered statistically significant.

## Results

Seventy high myopic eyes of 35 subjects were analyzed. The mean age was 26.42 ± 6.95 years (range 18 to 39 years). The mean spherical equivalent, axial length, CA, and total astigmatism were −8.08 ± 1.77 D (range-6.00 to-15.00D), 26.86 ± 1.04 mm (range 24.24 to 29.84 mm), -1.17 ± 0.64D (range −0.20 to −3.30D), and −0.83 ± 0.65D (range 0.00 to −2.50 D), respectively.

Of the 70 subjects, 36 eyes were classified as high astigmatism (≤ − 1 D of CA; mean-1.65 ± 0.49D), and 34 eyes were classified as normal astigmatism (> − 1D of CA; mean-0.67 ± 0.28D). Characteristics of the two groups are listed in Table 1, no significant differences were found for age, sex, axial length and spherical equivalent between two groups. The distribution of ONH parameters and RNFL thicknesses were listed in Table 2.

**Table 1:** **Characteristics of the two groups (**$\bar{x}\;\pm \;s$**)**

	**Normal astigmatism**	**High corneal astigmatism group**	**Range**	P^*^
Age	28.64(8.01)	25.42(7.44)	18-39	0.099
Sex(male/female)	15 /19	16/20	**—**	0.978^**△**^
Axial length	26.84(1.23)	26.87(0.84)	24.24-29.84	0.916
Spherical equivalent	−8.06(2.20)	−8.10(1.28)	−6.00— -15.50	0.930
Spherical refraction	−7.83(2.13)	−7.53(1.32)	−5.50— -15.00	0.473
Total astighmatism	−0.49(0.44)	−1.15(0.67)	0.00— -2.50	**0.026**
Corneal astigmatism	−0.67(0.28)	−1.65(0.49)	−0.20— -3.30	**0.000**

* Independent *t*-test, *Δ χ2* test.

**Table 2:** **Comparisons of ONH parameters in different astigmatism groups (**$\bar{x}\;\pm \;s$**)**

	**Normal astigmatism group**	**High corneal astigmatism group**	P^*^
Disc area	1.70 (0.32)	1.95 (0.58)	**0.030**
Rim area	1.25 (0.30)	1.44 (0.34)	**0.019**
Vertical C/D ratio	0.44 (0.13)	0.41 (0.18)	0.423
Average C/D ratio	0.48 (0.13)	0.44 (0.16)	0.374
Cup volume	0.096 (0.09)	0.097 (0.10)	0.970

*Independent *t*-test, ONH indicates optic nerve head, C/D indicates ratio cup-to-disk.

**Figure 2:**
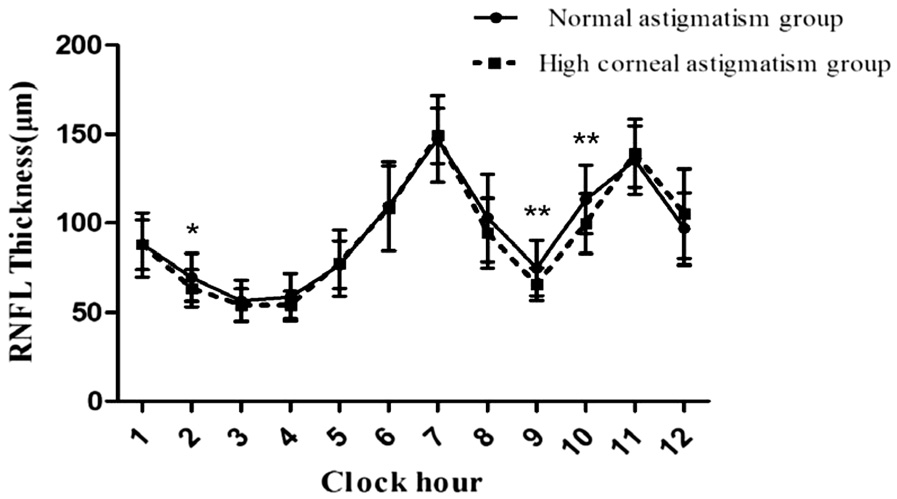
**RNFL profiles of normal astigmatism group (n = 34) and high CA group (n = 36).** Significant differences were found at 2 o’clock, 9 o’clock, and 10 o’clock, * indicates *P* < 0.05; ** *P* < 0.01.

Table 3 and Figure 2 showed the high CA group had significantly thinner RNFLs than the normal astigmatism group in the temporal, 2 o’clock, 9 o’clock and 10 o’clock sectors. The superotemporal and inferotemporal peak locations were farther temporally located in eyes with higher CA.

**Table 3:** **Comparisons of RNFL thickness and peak locations in different astigmatism groups (**$\bar{x}\;\pm \;s$**)**

	**Normal astigmatism group**	**High corneal astigmatism group**	P^*^
RNFL thickness (μm)			
Average	94.15 (8.59)	91.67 (7.25)	0.195
Superior	106.74 (12.50)	110.86 (13.72)	0.194
Nasal	61.79 (11.13)	57.50 (7.74)	0.067
Inferior	111.18 (16.34)	111.61 (13.95)	0.905
Temporal	97.06 (17.69)	86.75 (13.41)	**0.007**
RNFL peak location (degree)			
α angle	55.59 (8.86)	60.82 (8.34)	**0.013**
β angle	61.66 (7.20)	66.80 (10.74)	**0.043**
superotemporal peak	156.12 (19.37)	158.53 (22.51)	0.633
inferotemporal peak	167.32 (28.10)	167.11 (17.48)	0.970

* Independent *t*-test, RNFL indicates retinal nerve fiber layer.

Correlation analysis demonstrated significant correlations between CA and the nasal RNFL thickness, temporal RNFL thickness, α angle and β angle. The correlation coefficients for normal astigmatism were *r = −*0.316 (*P =* 0.008), *r = −*0.353 (*P =* 0.003), *r =* 0.452 (*P =* 0.000), *r = −*0.471 (*P =* 0.000), respectively, while correlation coefficients for high CA were *r = −*0.121 (*P =* 0.317), *r = −*0.102 (*P =*0.400), *r =* 0.250 (*P =* 0.037), *r = −*0.261 (*P =* 0.029), respectively (Table 4). There were no significant correlations between age, sex and ONH parameters, as well as between age, sex and ONH parameters RNFL thicknesses.

**Table 4:** Correlation analyses between CA and RNFL thickness / peak locations (Pearson analysis. n = 70)

	**Corneal astigmatism**		**Total astigmatism**	
	**r**	***P***	**r**	***P***
RNFL thickness				
Average	0.205	0.089	−0.044	0.716
Superior quadrant	0.220	0.068	0.191	0.113
Nasal quadrant	−0.316	**0.008**	−0.121	0.317
Inferior quadrant	0.035	0.774	−0.065	0.593
Temporal quadrant	−0.353	**0.003**	−0.102	0.400
RNFL peak locations				
α angle	0.452	**0.000**	0.250	**0.037**
β angle	−0.471	**0.000**	−0.261	**0.029**

RNFL indicates retinal nerve fiber layer.

## Discussion

The optical coherence tomographer is a modern imaging device designed to measure the RNFL and ONH parameters in a noncontact and noninvasive manner. RNFL measurements have been reliable and reproducible, and newer versions of optical coherence tomographers based on spectral domain technology that provide higher resolution and faster scanning speeds have been developed [8,9].

It has been reported that many factors, including refractive error, axial length, myopic optic disc tilt, eccentric scan location, and head tilt during the examination can affect the OCT measurements [10-13]. Lee *et al.*[14] reported that refractive error changes induced by wearing soft contact lenses of eight diopters without astigmatic power could affect RNFL thickness measured by a Cirrus HD OCT. They considered the RNFL thickness was underestimated in eyes with increasing negative refractive error, while it was overestimated with increasing positive refractive error. Therefore we hypothesize that, not only spherical refractive error, but also cylindrical refractive error can affect OCT measurements.

Our study showed that CA influenced spectral-domain OCT measurements of both RNFL thickness and ONH parameters. Eyes with higher CA had a larger disc area and rim area, thinner temporal RNFL thickness and farther temporally positioned superotemporal and inferotemporal peak locations of RNFL thickness. The high CA group had significantly thinner RNFL thickness than the normal astigmatism group in the 2 o’clock, 9 o’clock and 10 o’clock sectors (Figure 2).

Our results showed an intriguing finding that had not been reported previously. To date, the mechanism for changes in RNFL thickness and ONH parameters induced by astigmatism is not clear, however, possible explanations are as the followings: In high myopes, the optic disc is usually inserts obliquely. Once the optic disc tilts temporally, the nasal half of the optic disc elevates anteriorly, and the temporal half of the optic disc depresses posteriorly [15-17]. The CA may enhance the magnification effect among high myopes, which may be result in the disc area and distance from the disc rim border to the disk front surface were exaggerated. Such changes can lead to differences in reflectivity or backscatter detected by the OCT and subsequent differences in the RNFL thickness measurements.

These findings are ascribable to CA induced ocular magnification. The relationship between the measurement of the OCT image and the size of the actual fundus dimension can be expressed as ***t = p ·q ·s*** according to the *Littmann* formula [18], Where ***t*** is the actual fundus dimension, ***p*** is the camera magnification factor in the OCT imaging system, ***q*** is a magnification factor related to the eye, and ***s*** is the measurement in OCT. Various methods have been introduced to estimate factor *q* based on the ametropia, keratometry, and or axial length [19]. Although one can input the patient’s axial length and spherical equivalent in OCT, the effect of astigmatism has not been considered. Hwang *et al*[3] suggested that when the degree or axis of astigmatism changes, RNFL thickness measurement can be affected by changing the scan distance from the optic disc. All subjects in this study were WTR CA and the plus cylinder axis was within 30° of deviation from the 90° meridian. The maximum power was in the vertical meridian, the result for in the optic disc was vertically oval, and the scan circle was farther from the optic disc in the horizontal meridian. Thus, the measurement of RNFL thickness between two groups, using the same-sized scan circle, might be misleading because the RNFL thickness decreases at increasing distances from the optic disc [20]. There was a tendency for the RNFL thickness in the temporal and nasal regions to become thinner, even though the RNFL thickness of the nasal region was not statistically different between two groups (*P* = 0.067).

In this study, the sample size may be inadequate to reveal a statistically significant correlation between total astigmatism and the temporal / nasal quadrant average RNFL thickness. Further studies are needed to clarify this point.

In conclusion, we found that high corneal astigmatism with the rule influences the measurements of both RNFL thickness and ONH parameters by the Cirrus HD OCT. Eyes with higher corneal astigmatism had a larger disc area and rim area, thinner temporal RNFL thickness and farther temporally positioned superotemporal and inferotemporal peak locations of the RNFL in high myopes. Therefore, the degree of corneal astigmatism with the rule influences should be considered when interpreting the ONH parameters and peripapillary RNFL thickness measured by the Cirrus HD OCT in high myopes.

## Competing interests

The authors declare that they have no competing interests.

## Authors’ contributions

LL participated in the study design, reviewed the literature, collected the clinical data, and drafted the manuscript. JZ provided the conception and design of the study and reviewing the manuscript. HH collected the clinical data and selected the material. J-gY took part in the study design and performed the statistical analysis. S-rC participated in collected the clinical data. All authors have read and approved the manuscript.
